# Student perception of group dynamics predicts individual performance: Comfort and equity matter

**DOI:** 10.1371/journal.pone.0181336

**Published:** 2017-07-20

**Authors:** Elli J. Theobald, Sarah L. Eddy, Daniel Z. Grunspan, Benjamin L. Wiggins, Alison J. Crowe

**Affiliations:** 1 Department of Biology, University of Washington, Seattle, Washington, United States of America; 2 Department of Biological Sciences, Florida International University, Miami, Florida, United States of America; 3 School of Life Sciences and Center for Evolution and Medicine, Arizona State University, Tempe, Arizona, United States of America; University of North Carolina at Chapel Hill, UNITED STATES

## Abstract

Active learning in college classes and participation in the workforce frequently hinge on small group work. However, group dynamics vary, ranging from equitable collaboration to dysfunctional groups dominated by one individual. To explore how group dynamics impact student learning, we asked students in a large-enrollment university biology class to self-report their experience during in-class group work. Specifically, we asked students whether there was a friend in their group, whether they were comfortable in their group, and whether someone dominated their group. Surveys were administered after students participated in two different types of intentionally constructed group activities: 1) a loosely-structured activity wherein students worked together for an entire class period (termed the ‘single-group’ activity), or 2) a highly-structured ‘jigsaw’ activity wherein students first independently mastered different subtopics, then formed new groups to peer-teach their respective subtopics. We measured content mastery by the change in score on identical pre-/post-tests. We then investigated whether activity type or student demographics predicted the likelihood of reporting working with a dominator, being comfortable in their group, or working with a friend. We found that students who more strongly agreed that they worked with a dominator were 17.8% less likely to answer an additional question correct on the 8-question post-test. Similarly, when students were comfortable in their group, content mastery increased by 27.5%. Working with a friend was the single biggest predictor of student comfort, although working with a friend did not impact performance. Finally, we found that students were 67% less likely to agree that someone dominated their group during the jigsaw activities than during the single group activities. We conclude that group activities that rely on positive interdependence, and include turn-taking and have explicit prompts for students to explain their reasoning, such as our jigsaw, can help reduce the negative impact of inequitable groups.

## Introduction

There are broad, national calls for increased active learning in Science, Technology, Engineering, and Math (STEM) classes [1–3]. This call for higher structure and more student engagement in university STEM classrooms has placed a large emphasis on group work–particularly small, informal groups [4–6]. This emphasis on group work is driven largely by the observation that collaborative groups (as opposed to competitive groups or individual learning) increase learning, cultivate positive attitudes toward science, and enhance social identity as a scientist in STEM classrooms from primary school through university [7–11]. However, to reap these benefits, groups must be high functioning, i.e. those in which teammates collaborate, participate equitably, and disagree productively. Specifically, from pre-school [12] to university courses [6], students in high-functioning groups, learn more, report more positive attitudes toward science, and are more likely to identify as a scientist than their peers in lower functioning groups.

Teamwork is not only important in the classroom but is also critical in the STEM workplace. As products and tasks become increasingly complex, requiring expertise from a variety of people with a variety of skills, teamwork has become more prevalent than ever before [13]. Because of this, employers are more frequently looking for job candidates with a demonstrated ability to work well in teams [14,15]. For example, in a survey of 260 organizations from a variety of fields and industries, employers rated teamwork as the most important quality in recruits [15].

However, groups often operate at suboptimal efficiency or devolve into poorly functioning groups. Dysfunctional groups can happen for a variety of reasons including lack of formal training for group members in how to work effectively together [7], lack of formal training for instructors and team managers in how to effectively structure and manage groups [16,17], and lack of adequate tasks, e.g., tasks that are either loosely structured or insufficiently complex [13,18]. Thus, when implementing group work in any setting, managers, instructors, and others are left with the question: how do small, informal groups function and do dysfunctional groups impact individual performance?

Inequitable participation between group members is one form of dysfunctional group work that may be particularly damaging because it stunts team productivity in the workforce [19] and in STEM classrooms. Although inequitable group work can positively (or neutrally) impact some students, it frequently negatively impacts others [18,20]. Upon analyzing partner peer discussions in a large introductory university astronomy class, James and Willoughby [21], concluded that nearly 20% of the conversations students had about course content after a clicker question were unproductive either because students deferred to one student’s answer or because the students did not interact at all. Furthermore, Bianchini [22] found that 11 and 12 year old students who talked more learned more, but that the students who talked more were also more popular and/or perceived to have higher academic ability. Thus, the inequitable group participation perpetuated social status and disproportionately helped high status individuals; this pattern has also been demonstrated among university students [20]. In sum, inequitable participation can be detrimental to student learning, social belonging, and success by perpetuating stereotypes or reinforcing personal biases [18].

This kind of disproportionate disadvantage could be particularly detrimental to individuals who are numerical minorities in groups [23–25], and from backgrounds that are under-represented in STEM fields [26–28]. Specifically, as reviewed in Thompson and Sekaquaptewa [23], the performance of women and black students and employees, of any age, tends to be lower when they are the only woman or black person in their groups. This effect is exacerbated when negative stereotypes about particular demographic groups are related to the task being performed. For this reason, it is important to dissect overall trends into how different student populations respond to activities [29] and to continue investigating the impact on individual success of equitable participation and a student’s comfort in a group in the college classroom.

To understand group dynamics and the impacts on individual students, we asked our students a series of questions, which arose from student interviews wherein students shared their group work experience after various in-class activities [30]. For reasons described above, we were particularly interested in questions relating to equitable groups, perceived comfort in groups, and whether students worked with friends in their groups. We asked students these validated questions after two distinct types of group work: 1) a loosely structured task and 2) an intentionally-structured task that relied on student interdependence and individual accountability. We used the answers on these questions as predictors of student performance on content quizzes and then dissected the survey answers to determine if different sub-groups of students were perceiving group work similarly. Specifically, we asked three discrete questions: 1) how does reporting a dominator, being comfortable in a group, or working with a friend help or hinder individual content mastery? 2) which students are most likely to report working with a dominator, being comfortable, or working with a friend? 3) are activities structured to encourage positive interdependence better at facilitating group equity?

## Methods

### Ethics statement

This project was approved by the University of Washington Institutional Review Board and conducted under Human Subjects Division Application #44438. All students in the course completed the surveys and in-class activities as part of normal course work. Per the University of Washington Institutional Review Board, students were provided the opportunity to have their data removed from the study after being informed that a research study was being conducted in their class and that their data would be analyzed as part of the study. Students could additionally opt out of the study at any time by filing the request at a central office. Thus consent was informed and implicit if students did not opt to remove their data.

### Data sollection

#### Setting

We investigated these issues in an introductory biology course (the second course of a three-course sequence) at the University of Washington, a large R1 public university in Seattle, Washington, USA. The course was administered in winter quarter (January through March) of 2015. Content of the course included topics of cellular, molecular, and developmental biology; it met four times each week for 50 minutes and included a 2-hour weekly laboratory component. This course is required of biology majors and is predominantly composed of students who have not yet declared their major, instead indicating an interest in pre-science, pre-health, and pre-engineering. Students, primarily second year university students, but ranging from first year to fifth year university students (typically 18–24 years old), were grouped into one of two back-to-back sections, with 422 and 348 students in each, respectively.

#### Survey

To assess student perception of group work, we asked students a series of content- and response-processes-validated questions [31] in conjunction with the ASPECT survey (Assessing Student Perception of Engagement in the Classroom Tool). See [30] for details about the survey development and validation process, and for survey items. The ASPECT survey is specifically designed to gauge student perception of engagement in an active learning classroom during group work and is focused on three areas: value of group activity, personal effort, and instructor contribution. During the item validation process [30], two items related to group dynamics stood out as not aligning closely with any of these three areas. As we were interested in how small groups function, for this analysis we focused on these 2 items as well as a third item related to group composition (Table 1): 1) “Are you friends with at least one person that was in your group?” (binary: yes or no), 2) “One group member dominated discussion during today’s group activity” (6-point Likert scale, ranging from “strongly disagree” to “strongly agree”), and 3) “I felt comfortable with my group” (6-point Likert scale, ranging from “strongly disagree” to “strongly agree”). To limit our analysis to students who worked in groups of two or more, we also asked “how many other students did you work with on the activity?” Table 1 lists survey questions of interest and a summary of student responses to these questions. We implemented the survey three times over the course of the quarter in conjunction with group activities described below.

**Table 1:** Questions on the survey and the percentage of responses. Results are aggregated across all three iterations of the survey, so students’ views are repeated.

Question	Possible Answers	n Student Responses	% of Students Responding
Are you friends with at least one person that was in your group? (Eng2)	Yes	1022	78.6%
	No	278	21.4%
I felt comfortable with my group. (NewEng19)	Strongly Agree	502	38.6%
	Agree	589	45.3%
	Somewhat Agree	151	11.6%
	Somewhat Disagree	41	3.2%
	Disagree	13	1%
	Strongly Disagree	4	0.3%
One group member dominated discussion during today’s [topic] activity. (NewEng22)	Strongly Agree	97	7.5%
	Agree	195	15%
	Somewhat Agree	240	18.5%
	Somewhat Disagree	291	22.4%
	Disagree	391	30.1%
	Strongly Disagree	86	6.6%

#### Group activities

For three different topics taught on three different days over the course of the quarter, sections were randomly assigned to complete one of two types of in-class group activities: 1) “single-group activity” and 2) “jigsaw activity.” Importantly, the two activities were identical in terms of topic and content, but differed in how groups were structured and in how instructions specified how the learners should interact. For the single-group activity, groups worked sequentially through three sections of a worksheet. For the jigsaw activity, each student was given one of the three sections of the worksheet to work through independently, then conferred with students who mastered the same section, and finally formed a group of three with students who had completed the other sections of the worksheet to share and discuss all three sections. See [32] for more detail on activity design and an example of the activities. Note that this implementation does not match every jigsaw format in use [33], but is intended as a useful term to approximate the general structure of the activity.

To guide students to engage in the jigsaw groups, we embedded guiding prompts into the worksheet. These prompts explicitly structured group participation [34,35] by promoting dialogue, turn taking, and individual accountability [10,36]. Students in the single-group activity did not receive the guiding prompts.

Sections were assigned to one of the two group activity types on three different days over the course of the quarter: sections alternated activity types over the three implementations. In this way, we began to control for the fact that students self-registered into sections, and thus were not randomly assigned to treatment. For both types of activities, students worked in small groups, reporting that they worked with 2 (31.6% of students reported this), 3 (58.3%), 4 (8%), or more than 4 (1.8%) other people. Neither of the activities was graded or scored for completion: no course points were associated with completion of the activities.

#### Student performance

In conjunction with the survey and the activities, we also administered topic-specific, 8-question pre- and post-tests to assess students’ conceptual knowledge of the topic before and after each activity. The pre- and post-tests were identical for a given in-class activity and have been described previously [32]. Briefly, assessments were content and internal-structure validated [31]: test questions were iteratively revised based on feedback from four cell and molecular biology experts and Rasch analysis of post-test results confirmed that there was a range of item difficulty on each of the tests allowing discrimination between students. To ensure that the 8 items on each test were all measuring the same construct, we performed item-fit analysis (see [32] for further details of the validation process and the pre- post- assessments items themselves). Students took the pre-test the evening before the class session and took the post-test between the time class ended and before the class began the next day. We considered a student’s score on the post-test as ordinal. This eliminates the “ceiling effect” wherein a student who scores well on the pre-test has very little room for improvement compared to a student who students who scores poorly on the pre-test. Thus the interpretation of this model is “on average, the log-odds (converted to and reported as odds) of a student answering at least one additional question correct on the post-test are…”

#### Final dataset

The final dataset consisted of responses from three surveys, scores from three topic-specific identical pre- and post-tests, student characteristics sourced from the registrar’s office, and final grade in the course. Students were only included in the analysis if they completed at least two pairs of pre- and post-tests and the survey questions at least twice. This reduced our sample size from 776 students enrolled in the class to 684 students in the analysis. The final dataset is available as Supporting Information (S1 File).

### Data analysis

#### Mixed models and model selection procedure

Our study design required considering student responses as repeated measures: students responded three times to the survey and took the post-test three times. Thus, we fit mixed effects models, with student specified as a random effect. Including a student random effect accounts for the non-independence of the student-level responses, in other words, the fact that students took the survey multiple times [37].

To select the best-fit and most parsimonious model, we employed a backward model selection process. Specifically, we started by fitting a complex model, which included student characteristic fixed effects (from the registrar data: sex, ethnicity, and first generation status), course characteristic fixed effects (topic), and interactions (between the explanatory variable of interest and student characteristics). We then used backwards selection, eliminating terms that did not improve model fit. We assessed model fit using AIC (Akaike’s Information Criterion) and considered models with ΔAIC 2 or less to have equivalent fit; in these cases, we chose models with the fewest number of parameters [38]. Finally, we compared (and report) the ΔAIC of the final model compared to the null model (with only the student random effect); as a general rule, models with ΔAIC > 10 are a poor prediction of the data compared with the best model [38]. Once the best-fit model was selected, and if an ordinal variable was being tested as an explanatory variable (e.g., reporting a dominator), we tested if including this variable as ordinal or linear resulted in a better fit; in all cases, the qualitative results were similar when the explanatory variable was treated as continuous or ordinal. Below we describe the types of models we fit and the deviations from this model selection procedure. All models were fit in R version 3.3.2 [39].

#### Specific models and deviations in selection techniques

Question 1: How does reporting a dominator, being comfortable in a group, or working with a friend help or hinder individual content mastery?

We addressed this question by fitting four separate ordered logit mixed models (i.e., Cumulative Link Mixed Models or CLMM; in the ordinal package in R; [40]), considering a student’s score on the 8-question post-test as a categorical ordinal outcome. The first of the four models included all three covariates: reporting a dominator, being comfortable, and working with a friend. We built three subsequent models that included each covariate individually to test the hypothesis that these factors have independent effects on student performance. Fitting four models in this way explicitly tests the hypothesis that the three covariates of interest are individually influential on content mastery whether controlling for the others or not.

We controlled for possible confounders by including a suite of covariates in the starting model. First, we included students’ pre-score in the model to compare students with equivalent starting scores. Second, because this study was implemented on three different days during the quarter, different topics were covered on the pre-/post-tests (specifically eukaryotic gene regulation, PCR, and the cell cycle). We controlled for topic by including a dummy variable for topic in the model. Third, we used the three questions, did one student dominate your group, how comfortable did you feel in your group, and did you have a friend in your group, as possible predictors of content mastery. Finally, because each student comes to the classroom with a unique set of prior experiences and characteristics that may impact their content knowledge and mastery, we included demographic characteristics as predictors in starting models.

Question 2: Which students are most likely to report working with a dominator, being comfortable, or working with a friend?

To determine which students report a dominator, being comfortable, and working with a friend, we fit all models with student demographic characteristics. The model selection procedure thus tested the hypothesis that students with different identities differentially reported a dominator, being comfortable, and working with a friend.

Dominator: To determine which students more strongly agree that someone dominated their group, we fit ordered logit mixed models (CLMM; in the ordinal package in R; [40]), with the student’s response to the dominator question as the response. We modeled the data so that a “high response” indicates strong agreement that someone dominated the group.

Comfort: To determine which students more strongly agree that they felt comfortable in groups, we again fit ordered logit mixed models (CLMM; in the ordinal package in R; [40]), with the student’s response to the comfort question as the response. We maintained that order in our models so that a “high response” indicates strong agreement that the respondent felt comfortable in their group.

Friend: To determine which students are most likely to report working with a friend, we fit binomial mixed models. There is little variation in this outcome: 78.6% of students reported working with a friend, so fully complex models (as above) would not converge. Instead, we fit simpler models, omitting parameters until convergence was reached. Once a complex model converged we proceeded with model selection to find the simplest model that explained the variation in responses. We tested several starting models and regardless of the starting model, the result of the model selection procedure was identical.

Question 3: Are activities structured to encourage positive interdependence better at facilitating group equity?

The final model predicting which students are the most likely to report a dominator (Question 2 above) included the effect of the activity type. Thus, to answer the question we interpreted the final model described above.

## Results

The characteristics of students participating in the study were as follows: sex (male 33.5% and female 66.5%), ethnicity (combined into non-international-white (46.5%), Asian (35.5%), URM (11.3%; under-represented minority, including African American, black, Latinx, and Pacific Islander), and International (6.7%; the majority of the international students at the University of Washington come from the China, Korea, and India), first generation status (46.8%; reported to the registrar as whether or not a student’s parents have obtained a baccalaureate degree).

Question 1: How does reporting a dominator, being comfortable in a group, or working with a friend help or hinder individual content mastery?

**Fig 1:**
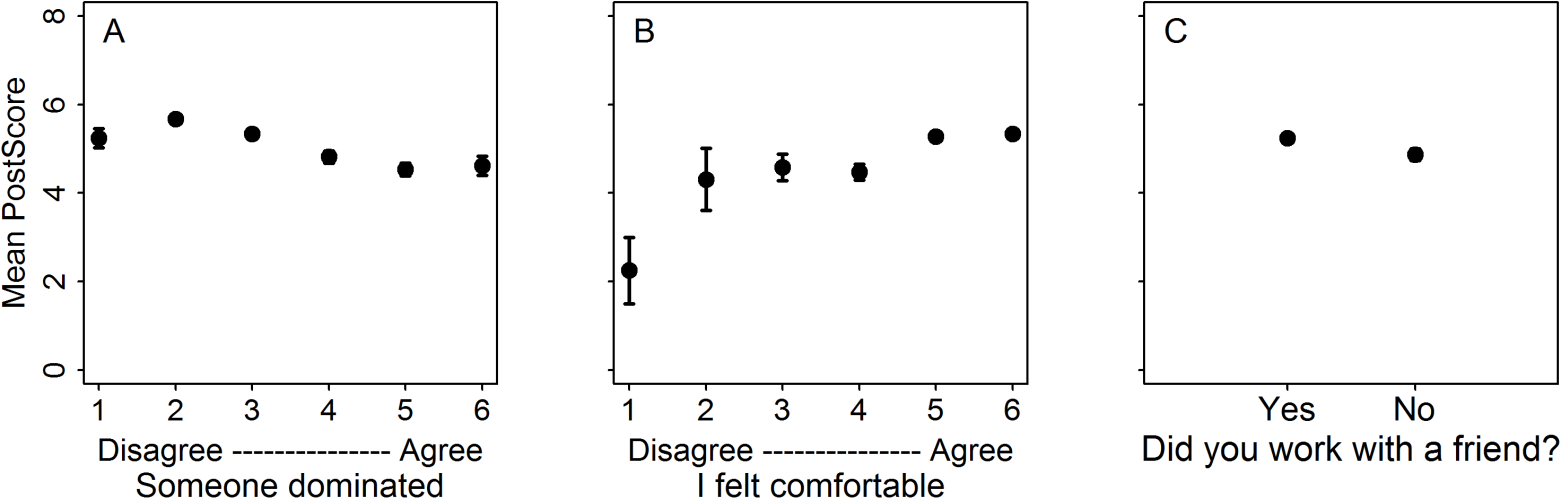
Raw means showing student performance on the post-test as a function of reporting a dominator, being comfortable in their group, and working with a friend. A) Controlling for pre-score, students who strongly agree there was a dominator in their group performed worse on the post-score than students who reported low levels of a dominator; B) Controlling for pre-score, students who report being comfortable in their groups score higher on the post-test; C) There is no effect of friend on performance (note that there is a difference in post-score, not controlling for pre-score wherein students who work with a friend score higher on the post-test (t = -2.6, p<0.05); this difference is noticeable in this figure). Error bars indicate standard error. Students answered the dominator and comfort questions on a 6-point Likert scale, from Strongly Disagree (1) to Strongly Agree (6). See Table 2 for final models and modeled estimates.

We asked whether factors that influence a student’s experience during group work are related to their understanding of the material. We found that, controlling for pre-score and topic, students who more strongly agreed that someone dominated their group performed worse on the post-test than when they less strongly agreed that they had a dominator in their group (Table 2, Fig 1). Specifically, the probability that a student scored at least one point higher on the post-test was 17.8% lower when a student more strongly agreed that someone dominated their group (Table 2). We note that on the 8-point post-test a one-question differential represents a 12.5 percentage point change in overall grade on the post-test. Similarly, when a student more strongly agreed to being comfortable in their group, they performed better than when they reported lower levels of comfort (Table 2, Fig 1), controlling for pre-score and topic. The probability that a student scored at least one point higher on the post-test is 27.5% higher when a student more strongly agreed to being comfortable compared to when they less strongly agreed to being comfortable (Table 2). There was no impact of working with a friend on student performance (Table 2). Including all of these factors together or individually in separate models did not qualitatively impact the effect of each factor (Table 2).

**Table 2:** Final models and associated estimates for predicting students’ performance on the post-test and which students report a dominator, being comfortable, and working with a friend. Coefficients are presented as odds (transformed from logodds); models were fit as cumulative link mixed models (postscore, dominator, comfort) or logistic regression (friend; see methods for details). Bolded coefficients represent statistical significance to α = 0.05. Grey cells indicate variables that were not included in the full model, empty cells indicate variables that were included in the full model and then were dropped during the model selection process. Superscripted notes indicate starting models and additional notes.

Outcome	Pre-score	Dominator^1^	Comfort^1^	Friend	Sex^2^	Ethnicity^3^	First Gen.	Trt^4^	Grade^5^	ΔAIC^6^
***Performance***										
Postscore^7^^,^^8^	**1.92**	**(-)1.17**	**1.27**						^10^	423.96
Postscore^7^^,^^9^	**1.92**	**(-)1.18**								413.15
Postscore^7^^,^^9^	**1.95**		**1.28**					**1.27**		414.16
Postscore^7^^,^^9^	**1.96**							**1.27**		402.55
***Who Reports Group Characteristics***										
Dominator^10^						URM 1.40		**(-)1.67**	**(-)1.42**	57.43
						**AA 1.69**				
						**Int 5.87**				
Comfort^10^				**5.25**						90.57
Friend^10^^,^^11^										0

^1^Included in the initial model as ordinal categorical, retained in the final model as continuous

^2^Reference group: Female

^3^Reference group: white students; abbreviations: AA = Asian American, URM = Under-represented minority (see methods for details), Int = International students (see methods for details)

^4^Reference group: Single-group activity

^5^Final Grade in course

^6^Change from null model: Outcome ~ 1 + (student random effect); Models were selected using backwards selection, starting with the most complex model

^7^Controling for topic

^8^Outcome (Postscore) ~ Performance (Prescore) + Sex + First Generation Status + URM + Dominator + Friend + Treatment + Topic + (student random effect) (note: there were no interactions in the initial model)

^9^Outcome (Postscore) ~ Performance (Prescore) + Demographic Characteristics (Sex + First Generation Status + URM) + Dominator OR Friend OR Treatment + Topic + Interactions between Dominator, Comfort, OR Friend*Demographic Characteristics + (student random effect)

^10^Outcome (Dominator OR Comfort OR Friend) ~ Performance (Course Grade) + Demographic Characteristics (Sex + First Generation Status + URM) + (the two that are not the outcome of Dominator, Friend, or Treatment) + Topic + Interactions between Treatment *Demographic Characteristics + (student random effect)

^11^Null model selected as best fit model.

Question 2: Which students are most likely to report working with a dominator, being comfortable, or working with a friend?

**Fig 2:**
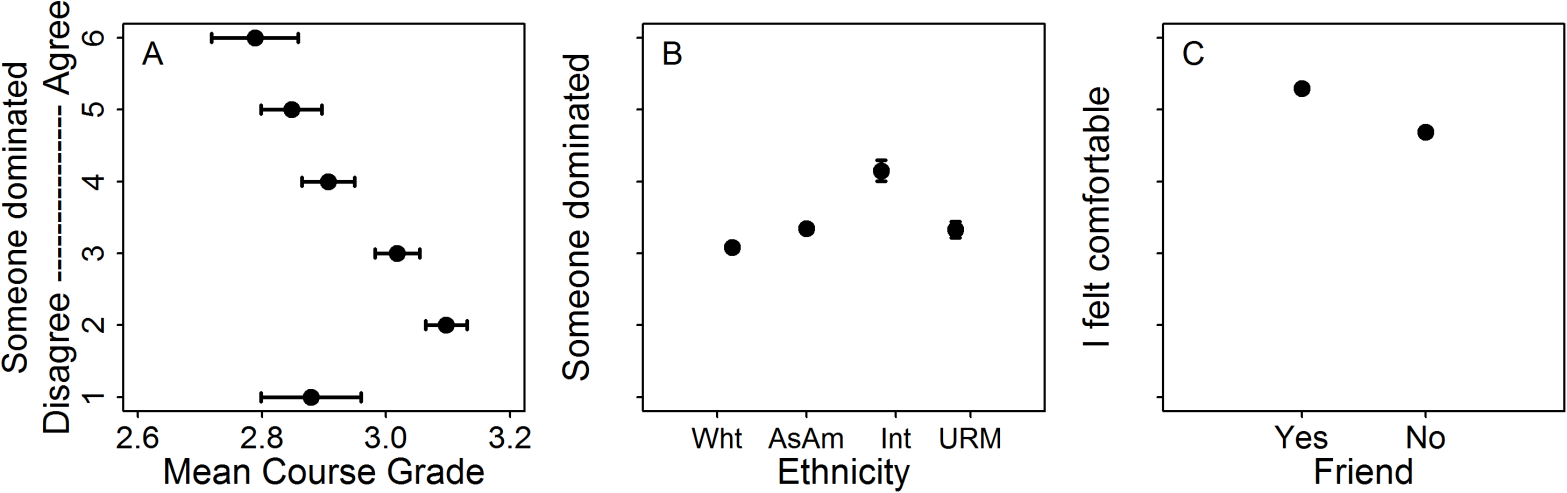
Raw means show that some student characteristics predict which students report a dominator and being comfortable in their group. A) Students who have low course grades report higher levels of a dominator; B) International students (Int) and Asian-American students (AsAm) report higher levels of a dominator than white students (Wht), and there is no difference in dominator report rate between white students and URM students; C) The single predictor determining a student’s comfort in their group was whether (or not) they worked with a friend. Error bars indicate standard error and are present on all plots, despite being very small and subsumed by points in some cases. See Table 2 for final models and modeled estimates.

To assess how different students perceived the in-class group activities, we asked whether student characteristics, or activity type predicted their perception of group dynamics. Controlling for topic, international students and Asian-American students were more likely to report a dominator than white American students (Table 2, Fig 2), all else equal. Specifically, an international student was 487% (i.e. 5.9 times) more likely to report a dominator than white American students, and the probability that an Asian American student reported a dominator was 69% higher than for white students (Table 2), all else equal. Additionally, students with higher course grades were 42% less likely to report a dominator than students with lower course grades. Controlling for topic, the single variable that predicted whether students reported being comfortable in their group was whether or not they reported working with a friend (Table 2, Fig 2). Students who worked with friends were 425% (i.e. 5.25 times) more likely to report being comfortable compared to students who did not work with a friend (Table 2). Finally, there was no discernable difference, in terms of ethnicity, first generation status, or course grade, between students who worked with a friend and who did not; our model selection procedure selected the null model every time (Table 2).

Question 3: Are activities structured to encourage positive interdependence better at facilitating group equity?

**Fig 3:**
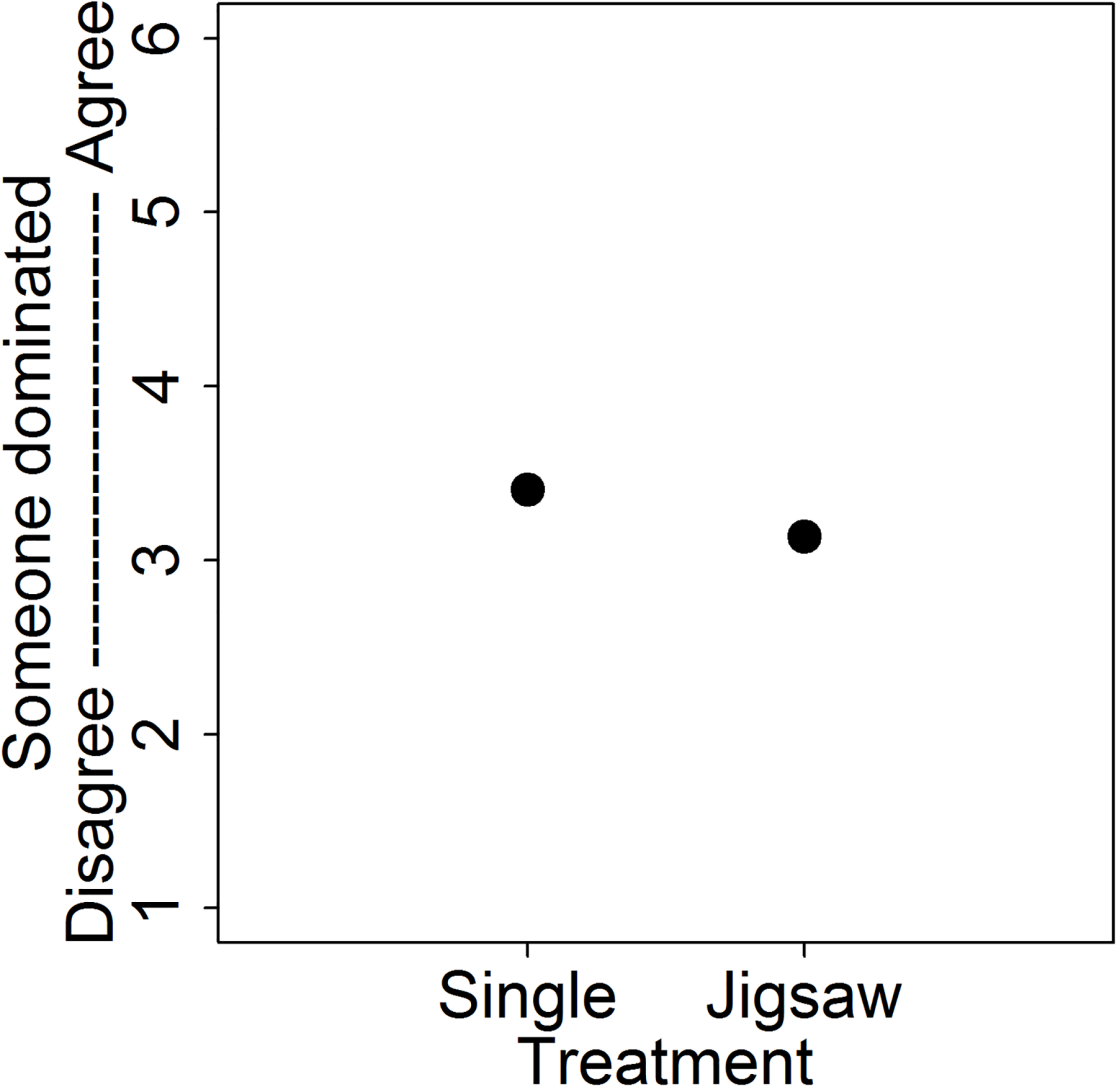
Raw means show that a carefully designed jigsaw in-class activity reduced the dominator report rate compared to a single-group activity. Error bars, which are present but subsumed by the point, indicate standard error. See Table 2 for final models and modeled estimates.

Given that student performance was enhanced on the jigsaw activity [32], we wondered if student perception of equitable participation may also be enhanced on the jigsaw activity. This is plausible because the jigsaw activity was explicitly structured to maximize student interdependence and collaboration, whereas the single-group activity lacked this structure. We compared student perception of group equity after completing the jigsaw activity and after completing the single-group activity. We found that, all else equal, students were 67% less likely to agree that someone dominated their group during jigsaw activities than on the single-group activity (Fig 3, Table 2).

## Discussion

We sought to understand how the composition of small groups and the student interactions in these groups impact student performance in a university introductory biology classroom. We found that students demonstrate lower content mastery (measured by a post-test) when agreeing more that someone dominated their group than when agreeing less that someone dominated their group (Fig 1), and that students demonstrate higher mastery when agreeing more to being comfortable in their groups than when agreeing less to being comfortable. Finally, we show that during a carefully constructed jigsaw activity, students were less likely to agree that someone dominated their group (Fig 2). This suggests that jigsaw activities, with intentional structure more effectively promote equity than group activities with less intentional structure.

### Eliminate dominators and promote comfort in groups

Students who dominate group conversations and group interactions have been documented in STEM classrooms ranging from Physics and Astronomy [21] to Biology [41] to Engineering and Math [42,43], as well as across all sectors of the STEM workforce [13]. All of these studies have a key attribute in common: one group member was more likely to dominate when the assignment was high-stakes, meaning that it was graded, would be evaluated by a boss, or would impact promotion, and thus aligned with personal incentives [13,18,21,43]. In contrast, the assignments implemented in this study were low-stakes and not scored for completion or accuracy (although they were collected). However, at two times during the activity, students were asked 3–4 content-based clicker questions which were scored for accuracy and assigned a very small number of course points. These clicker questions may have contributed to the rates of dominators reported in groups, despite the activity itself not being tied to course points. Either way, our data suggest that dominators can influence group function in a negative way, even for low-stakes tasks.

The finding that Asian-American students and international students report a dominator at higher rates than white students could be related to individual students’ learning preferences: as Eddy et al. caution [29], not all students experience group work the same way. For example, there is some evidence that Western-developed student-centered practices such as cooperative learning have not been equally effective when implemented in Asia due to different cultural values [44]. Second generation Asian American students also showed decreased performance when asked to talk while learning a new task, whereas talking improved performance by white students and students from Western cultures [45,46]. In this way, if one student dominates a conversation, it may be particularly jarring to students from cultural backgrounds that place more emphasis on introspection and thinking on one’s own as opposed to a direct relationship between talking as a way to work through ideas. Similarly, students with low-course grades are more likely to agree that someone dominated their group than students with high-course grades. This tendency may be related to lower-performing students desiring additional time to work through content on their own before joining a group. Alternatively, lower-performing students may lack the confidence to challenge their peer’s ideas, leading to one group member dominating the discussion. Overall, our results suggest that certain students are systematically more likely to underperform in classes where dominators persist in group work.

It is important to note that dominators may self-identify. During think-alouds and focus groups (see [30] for details), it became clear that students did not always perceive dominators as negative. Some students contended that, “I think that the person who knows well should be leading the group activity” and others reported “I feel like I dominated a lot of the discussion.” We did not ask students whether or not they were self-identifying as the dominator, so we cannot make the comparison between students who felt dominated and students who themselves dominated discussion. Exploring this difference in group roles would be an interesting future avenue of research.

Comfort was strongly positively correlated with student performance; in fact, reporting being comfortable had an even larger impact on student performance than reporting a dominator (Table 2). It is interesting to note that the vast majority of students agreed that they feel comfortable in their group (Table 1). The single factor that predicted students’ comfort was whether or not they worked with a friend. However, reporting working with a friend was not directly correlated with performance. Similarly, it was not possible to determine which students worked with a friend because almost all students (78.6%) said they worked with a friend. Taken together, this stresses the importance of instructors working toward increasing students’ comfort in groups. Comfort can also be thought of as “safety” wherein groups are safe places for individuals to share ideas [47,48] and to be themselves [49]. Functionally, this is a different task for instructors than demonstrating the safety of the classroom environment or of the instructor-student relationship. Instructors who can best convince their class that their peers are essentially ‘on their team’ will facilitate more complete learning. Intentional strategies that may increase comfort in group work include 1) establishing group norms [19], 2) having many opportunities to perform group work, ideally with groups that are more stable than single class periods (thus practice the skill of working together while getting to know each group member personally; [13,18,19,47,48]), 3) using approaches to help students get comfortable with each other in class (e.g., allowing for social connections within groups; [19,47,48]), or 4) allowing students to self-select into groups so that they can choose to work with friends if they wish [49,50].

### Group work

We wondered if certain group activities could negate some of the negative effects of dominators or increase student comfort in groups, in part because student performance was enhanced on the jigsaw activity [32]. We found that, compared to the single-group activity, a jigsaw activity dramatically decreased (by nearly 70%) the likelihood of students more strongly agreeing that someone dominated their group (Table 2, Fig 2). However, the jigsaw activity had no impact on students’ perceived comfort in groups.

Our findings underscore the importance of constructing group work activities so that all students can succeed–specifically by minimizing the chance that one student dominates and maximizing the chance that students are comfortable. To accomplish these goals, we, like others (e.g., [10,18,29,51–53]), offer suggestions. Improved group function is more likely when the task is appropriately complex and requires input from many individuals [7,13,18,53–56], and when group members experience interdependence as opposed to independence [13,56,57]. Interdependence has the additional benefit that it can help decrease competition between groupmates [9], which is known to disproportionately hinder equity as perceived by women and minority students [58,59]. The jigsaw activity implemented in this study is one example of an activity that is appropriately complex and encourages interdependence.

A common way to increase interdependence between students is by increasing structure in the group work [33] and that structure can change either *how* groups perform or *what* groups perform. Two major ways to influence how groups perform are to encourage groups to establish norms of behavior and operation [19,60] or to assign individual roles to group members [18]. For example, in an observational study of how effective teams function at a large technology firm, Duhigg [19] found few consistencies other than that high-performing groups had clear group norms.

Furthermore, one way to change how groups perform is by assigning individual roles to individual students [18]. When the task requires input from each role, this is a natural way to create interdependence between students. We caution that assigning group roles is not the same as assigning groups. Although there is some evidence that assigned groups can benefit some learners [61], there is evidence that self-selected groups are best for students from marginalized and minority groups. For example, Cooper and Brownell [49] found that group work increases the pressure on LGBTQIA students (lesbian, gay, Bisexual, transgender, queer, intersex, and asexual) to explain or defend their sexual identity, decreasing their comfort in class, and potentially affecting their performance. However, these same students with non-normative gender identities felt more comfortable in self-selected groups [49]. Similarly, Freeman et al. [50] found that students self-selected into groups with students who were similar to them, first based on appearance (ethnicity and gender) and then by performance.

A carefully constructed activity can also provide structure to *what* students are doing. For example, there is evidence suggesting that students explain their reasoning more when instructors include explicit prompts (as simple as “explain your reasoning”) on the assignment [41]. Thus it follows that with explicit prompts geared toward interdependence, such as “take turns,” “defend your answer,” and “probe your groupmates for justification,” students may work together more effectively and equitably, though this should be rigorously tested.

### Limitations and future directions

Our data do not provide an explanation for *why* the jigsaw activity resulted in fewer dominators. Thus, we are left with complementary, albeit distinct, hypotheses to explain the patterns we observed: 1) interdependence and self-accountability are key to promoting group equity, or 2) activity logistics and explicit prompts are the key to promoting group equity. By design, highly-interactive activities, such as jigsaws are thought to be at the apex of student cognitive engagement and student learning (see the ICAP framework, [62]) because these activities promote interactive dialogue, co-constructing knowledge, and interdependence. Jigsaws specifically stimulate interdependence because students first become experts then work collaboratively with other student-experts to complete a task. However, the jigsaw activity tested here also had explicit prompts to guide students through the activity. Perhaps it was these prompts, which promoted turn-taking and guided students to act as facilitators not lecturers which aided group equity. Further research testing the synergy or disparateness of these two hypotheses is warranted as a guide to informing classroom instruction.

Another limitation is that we aggregated data from different demographic groups in order to obtain sufficient statistical power to rigorously test our hypotheses. For example, all African-American, black, Latinx, Pacific Islander, and Native American students were combined into a single group termed “under-represented minority” students. Similarly, we combined all Asian-American students whose heritage span all Asian countries and “international” students who encompass all students who are not citizens of the USA. Similarly, we considered students’ sex instead of gender, which is more fluid than the two definitive categories the registrar’s office currently offers: male and female. We recognize that students come from different cultural and social backgrounds so bring unique experiences that impact how they perceive group work [63]. Future studies could seek to disaggregate these groups and test if the observed patterns hold, and whether particular groups of students are driving the patterns. Additionally, qualitative techniques such as one-on-one student interviews or focus groups could provide insight into the range of ways different assignment structures might impact perceptions of equity.

Finally, we did not identify which students worked together in groups, or if students were self-identifying as the dominator. By adding these layers of information, it could be possible to determine how well dominators perform relative to their groupmates and how aware students are of their own behavior. Additionally, if we knew who was working with whom we would be able to determine if a student’s solo status in a group (i.e. being the only person in their group with a particular racial or gender identity) impacts their perception of inequity. Finally, by asking students if they are self-reporting as the dominator we could clarify how to target interventions to reduce the prevalence of a dominator. Do we first need to make students aware that their actions are not promoting turn-taking, or will simply encouraging more equitable participation be sufficient? Our data suggest that group equity and comfort in a group are correlated with individual performance, but further studies into how exactly groups function are merited.

### Conclusions

Maximizing functional groups by promoting comfort and discouraging dominators can improve student learning. Here we report that jigsaw group activities can be one way to accomplish these goals. Providing students with opportunities to work collaboratively in high-functioning groups may have the additional benefit of preparing students for the workforce. Specifically, employers rate teamwork as the highest-rated, most desirable recruit attribute, but they also think that new college graduates have fewer teamwork skills now compared to previous recruits [15]. Thus, by continually practicing group work in high-functioning teams, instructors can achieve higher student outcomes and potentially better-prepare students for the STEM workforce [13,18].

## Supporting information

S1 File Data used in this study. (CSV)
